# Evaluating the Impact of Index Metastasis Resection in Patients with Multiple Brain Metastases

**DOI:** 10.3390/cancers17203281

**Published:** 2025-10-10

**Authors:** Maria Goldberg, Luisa Mona Kraus, Cvetina Vatcheva, Denise Bernhardt, Stephanie E. Combs, Chiara Negwer, Bernhard Meyer, Arthur Wagner

**Affiliations:** 1 Department of Neurosurgery, School of Medicine, Klinikum Rechts Der Isar, Technical University Munich, 81765 Munich, Germany; 2 Department of Radiation Oncology, School of Medicine, Klinikum Rechts Der Isar, Technical University Munich, 81765 Munich, Germany; 3 German Cancer Consortium (DKTK), Partner Site Munich, 80336 Munich, Germany; 4 Institute of Innovative Radiotherapy (iRT), Department of Radiation Sciences (DRS), Helmholtz Zentrum Munich, 85764 Munich, Germany

**Keywords:** neuro-oncology, brain metastases, neurosurgical resection

## Abstract

**Simple Summary:**

This retrospective study evaluated patients with single, oligometastatic, and multiple brain metastases who underwent surgical removal of a symptomatic “index” lesion followed by radiotherapy of the remaining metastases. The aim was to assess whether this combined approach improves survival and functional outcomes. The median survival did not differ significantly between groups, but patients with complete resection of the index lesion experienced fewer postoperative seizures. These findings suggest that while surgery plus radiotherapy may not extend survival in all patients with multiple brain metastases, carefully selected individuals can still achieve meaningful functional benefits. Clinically, this highlights the potential role of aggressive local therapy in selected patients, expanding options beyond purely palliative strategies.

**Abstract:**

Background: The impact of surgical resection on the survival and functional outcomes of patients with multiple brain metastases remains a critical question in neuro-oncology. Methods: This retrospective study involved 160 patients who underwent surgical resection of brain metastases from 2017 to 2020. Patients were classified by the number of metastases—single, oligometastatic, or multiple—and whether complete removal of the main symptomatic lesion was achieved. Outcomes such as survival rates, complications, and functional status were assessed. Results: Among the patients, 48.1% had a single metastasis, 21.9% were oligometastatic, and 30% had multiple metastases. Survival did not differ by number of metastases when the main lesion was completely resected and remaining lesions were radiated (*p* = 0.6172). Complete resection increased mean survival to 15.74 months compared with 4.5 months without it. Additionally, patients who underwent complete resection experienced fewer seizures (16.2% vs. 32.6%, *p* = 0.019), implying a lower seizure risk. Functional independence was maintained post-surgery. Conclusions: While multiple brain metastases are generally associated with poor outcomes, a trend toward longer survival was observed after complete resection of the index metastasis, though this was not statistically significant. Radiation of residual lesions remains important to support prognosis. Reducing the tumor volume is key to lowering seizure risk. This study supports the role of aggressive surgical interventions, paired with radiation, to potentially enhance outcomes in patients with multiple brain metastases.

## 1. Introduction

Brain metastases account for the most prevalent form of intracranial tumors, affecting 10–30% of adult cancer patients. As the landscape of neuro-oncology evolves, the role of surgical intervention in managing multiple brain metastases presents considerable therapeutic potential and challenges. Combining surgery, radiotherapy, and chemotherapy is now considered essential for optimal care [1,2,3].

Historically, radiotherapy dominated the treatment paradigm for brain metastases, offering a non-invasive approach to control intracranial disease [4,5,6,7]. However, advancements in surgical techniques have positioned surgical resection as a fundamental component of treatment, particularly for symptomatic relief and histopathological diagnosis [8,9,10,11,12]. Surgical resection is vital for managing acute neurosurgical conditions, such as hemorrhage or significant mass effects from large lesions, where timely intervention can significantly improve outcomes [13].

The benefits of surgical resection extend beyond immediate symptomatic relief. Studies have shown that removing the index metastasis can enhance the efficacy of radiosurgery and systemic therapies by reducing tumor burden [14,15]. Some authors claim that patients with two to three brain metastases, well-controlled systemic disease, a Karnofsky Performance Status greater than 60, and surgically accessible lesions can significantly benefit from surgical treatment, achieving survival and quality of life improvements comparable to those with a single metastasis, alongside reduced or resolved neurological deficits [16]. Surgical resection in this group has been shown to improve survival when coupled with adjuvant therapies, underscoring the importance of a tailored treatment approach [17]. Moreover, the integration of surgery into a multimodal treatment plan is supported by studies demonstrating that patients with limited systemic disease and favorable performance status benefit most from aggressive local therapies [18].

Emerging evidence highlights that the choice of treatment should be individualized, taking into account factors such as functional status, number and size of metastases, and systemic disease burden [16,19].

Our study seeks to further elucidate the role of surgical resection, particularly complete resection of the index metastasis defined as the clinically dominant lesion (symptomatic, largest, or causing significant mass effect/hemorrhage), alongside adjuvant therapies in patients with multiple brain metastases. This investigation aims to refine surgical strategies, improve survival outcomes, and enhance the quality of life for this patient population, contributing valuable data to the evolving treatment landscape for brain metastases.

## 2. Materials and Methods

### 2.1. Patient Selection and Data Collection

A single-center retrospective study was conducted involving 160 patients with histologically confirmed brain metastases, treated at our neurosurgical department from 2017 to 2020. All patients who underwent surgical resection for brain metastases at our institution were included. No patients were excluded other than those lacking postoperative imaging suitable for volumetric analysis. This study aimed to evaluate the impact of surgical resection of brain metastases and associated factors on patient outcomes, including survival, postoperative complications, and functional status. Data were collected using medical records extracted from our department’s computerized system. This included patient demographics, clinical characteristics, primary tumor histology, number and distribution of brain metastases, and treatment details. Patients were categorized based on the number of brain metastases—single, oligometastatic (2–3 lesions), and multiple (>3 lesions)—and whether complete resection of the resected metastases was achieved.

Postoperative outcomes such as Karnofsky Performance Status (KPS), seizure occurrence, and complications graded by the Clavien–Dindo classification [20,21] were documented. Additionally, treatment details, including adjuvant radiation and systemic therapies, were recorded.

### 2.2. Surgery

The decision for surgical intervention was guided by the interdisciplinary neuro-oncology panel, considering factors such as symptomatic lesions, mass effect, intratumoral bleeding, ambiguous diagnosis, and the presence of large posterior fossa tumors with a potential risk of herniation or hydrocephalus. Intraoperative frozen sections were obtained in all cases.

The surgical goal was maximal safe cytoreduction, supported by Brainlab navigation with 3T MRI T1-weighted and FLAIR sequences. Transcranial Magnetic Stimulation (TMS) mapping was employed preoperatively to map functional areas, and neuromonitoring with Motor Evoked Potentials (MEPs) was continuously applied throughout the surgical procedures. These advanced technologies and techniques facilitated effective tumor resection while minimizing the risk of damage to critical brain functions, ensuring patient safety and optimizing surgical outcomes.

### 2.3. Tumor Volumetric Analysis

Postoperative MRI evaluations were conducted within 72 h of surgery to assess and calculate tumor volumes. T1-weighted MRI sequences, with and without gadolinium contrast, were analyzed. Residual tumors contributing to any post-surgical signal changes were considered, and complete resection was defined as having a residual tumor burden of zero.

Volumetric measurements were executed manually using Origin^®^ software (Brainlab AG, Munich, Germany). The contrast-enhancing components of the tumors were segmented for precise assessments.

### 2.4. Statistical Analysis

Statistical analysis was conducted using IBM SPSS version 29.0.0. Descriptive statistics were calculated for all variables, with continuous data presented as the median and interquartile range (IQR) due to their non-normal distribution as determined by Shapiro–Wilk tests. Categorical variables were summarized as frequencies and percentages. Survival analysis was performed using Kaplan–Meier curves with log-rank tests to compare survival between groups. Non-parametric tests were employed for group comparisons, including the Mann–Whitney U test for comparing two independent groups and the Kruskal–Wallis test for comparing three or more groups. Associations between categorical variables were assessed using Chi-square tests, with Fisher’s exact test applied when expected cell counts were less than 5. For paired comparisons of pre- and postoperative measures, Wilcoxon signed-rank tests were utilized. Effect sizes with 95% confidence intervals were reported wherever feasible (odds ratios for categorical outcomes, Cliff’s delta for continuous data). Where effect sizes or confidence intervals could not be calculated due to methodological or sample size limitations, this is stated explicitly. Missing data were not imputed; analyses were performed with available cases only. Statistical significance was set at *p* < 0.05 for all analyses.

## 3. Results

### 3.1. Clinical Characteristics

The study cohort consisted of 160 patients with brain metastases who underwent surgical resection (Table 1). Males represented a slightly greater portion (54.4%, *n* = 87) compared with females (45.6%, *n* = 73). The median age was 67 years (IQR 56–74), ranging from 20 to 90 years. Most patients (83.1%, *n* = 133) had no history of epileptic seizures before surgery, while 16.9% (*n* = 27) presented with seizures preoperatively.

Regarding disease status, 60.0% (*n* = 96) of patients had synchronous extracranial disease, while 40.0% (*n* = 64) had metachronous disease. The most common primary tumor was lung cancer (33.1%, *n* = 53), followed by melanoma (15.0%, *n* = 24), gastrointestinal cancers (9.4%, *n* = 15), and breast cancer (8.8%, *n* = 14). Other histologies included kidney (7.5%, *n* = 12), cancer of unknown primary site (3.1%, *n* = 5), urothelial cancer (3.1%, *n* = 5), and other types (20.0%, *n* = 32).

Nearly half of the patients (48.1%, *n* = 77) presented with a single brain metastasis, while 21.9% (*n* = 35) had oligometastases (2–3 lesions) and 30.0% (*n* = 48) had multiple metastases (>3). The rate of complete resection of specifically targeted (resected) or index metastases was 83.8% (*n* = 134).

Most patients (76.9%, *n* = 123) did not experience adverse effects from surgery. Among the 37 patients with complications, the distribution according to Clavien–Dindo grading showed 35.1% (*n* = 13) with grade I, 5.4% (*n* = 2) with grade II, 2.7% (*n* = 1) with grade IIIa, 13.5% (*n* = 5) with grade IIIb, 18.9% (*n* = 7) with grade IVb, and 24.3% (*n* = 9) with grade V complications.

Adjuvant and neoadjuvant treatment included radiation therapy in 84.4% of patients (*n* = 135), mostly postoperatively (including several patients who received intraoperative radiation as part of the Lex-IORT study [22] [NO_PRINTED_FORM], and systemic therapy in 67.5% of patients (*n* = 108). Of the 70 patients with single metastases, 32 (45.7%) received postoperative Hypofractionated Stereotactic Radiotherapy (HFSRT), 11 (15.7%) received intraoperative radiation, and 27 (38.6%) received adjuvant radiation at other hospitals. Of the 65 patients with multiple brain metastases, 23 (35.4%) received HFSRT of the resection cavity and stereotactic ry (SRS) for the remaining metastases. Five (7.7%) patients received intraoperative radiation with SRS for the remaining metastases postoperatively. Fourteen (21.5%) patients received Whole-Brain Radiation Therapy (WBRT), and information for twenty-three (35.4%) patients was unavailable due to treatment at different hospitals. Of the 25 patients, 2 (8%) denied radiation therapy and the rest (92%) did not receive it due to their low functional status. At the time of analysis, 65.0% (*n* = 104) of patients were alive, while 35.0% (*n* = 56) were deceased.

Patients presented with a median of two brain metastases (IQR 1–4), with the number of lesions ranging from one to sixteen. The median total tumor volume was 14 cm^3^ (IQR 7–31). The median volume of specifically operated metastases was 12 cm^3^ (IQR 6–26).

Karnofsky Performance Score (KPS) remained relatively stable from pre- to postoperative assessment, with median values of 80% both preoperatively (IQR 60–90) and postoperatively (IQR 60–90). Overall, 113 of 160 patients (70.6%) maintained or improved their KPS, while 47 patients (29.4%) experienced a decline, most of which were <10 points. This finding is consistent with previous studies showing that surgical resection can maintain functional status in patients with brain metastases. The median length of hospital stay was 10 days (IQR 6–17), with individual stays ranging from 3 to 74 days; in absolute terms, 94 patients (58.8%) were discharged within 10 days, while 66 patients (41.2%) required longer hospitalization.

A comparison of survival in patients who achieved complete resection of index metastases and received adjuvant and neoadjuvant radiation, stratified by the number of lesions.

The Kaplan–Meier survival analysis examined differences in survival outcomes between patients who achieved complete resection of resected metastases and received adjuvant radiation therapy, stratified by the number of brain metastases. Patients with a single metastasis demonstrated mean survival of 17.70 months (SE = 2.64, 95% CI: 11.81–22.33). Those with oligometastases (2–3 lesions) showed mean survival of 22 months (SE = 4.75, 95% CI: 12.27–31.73), while patients with multiple metastases (>3) had mean survival of 12.14 months (SE = 3.09, 95% CI: 5.87–18.40).

**Figure 1:**
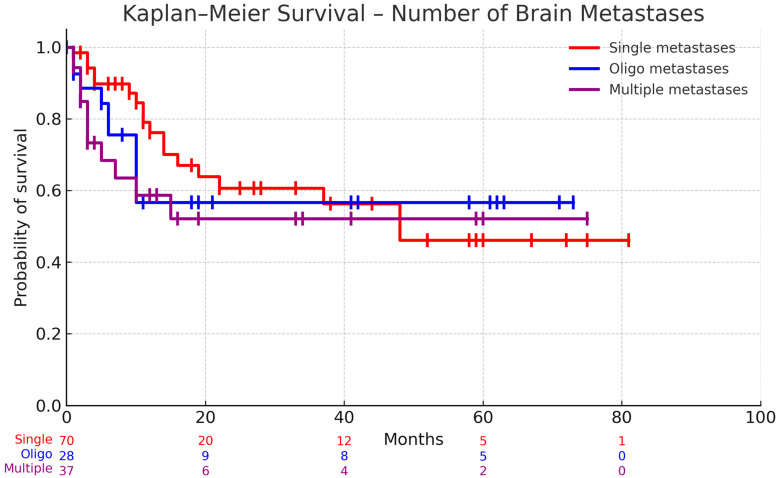
A comparison of survival based on the no. of metastases in patients where complete resection of index metastases was achieved and the patient received adjuvant radiation.

Statistical analysis revealed no significant difference in survival between these three groups (*p* = 0.6172), suggesting that in patients who achieved complete resection of metastases and received adjuvant radiation, the number of brain metastases alone did not significantly impact survival outcomes (Figure 1).

Kaplan–Meier curves are shown for patients with a single metastasis, oligometastatic disease (2–3 lesions), and multiple metastases (>3 lesions). Numbers at risk are displayed beneath the x-axis. Median survival was 48 months in the single-metastasis group, whereas the median was not reached in the oligometastatic and multiple metastases groups. Global comparison with the log-rank (Mantel–Cox) test was not significant (χ^2^ = 1.63, df = 2, *p* = 0.44). The log-rank test for trend was likewise non-significant (χ^2^ = 1.58, df = 1, *p* = 0.21). The Gehan–Breslow–Wilcoxon test showed a similar pattern (χ^2^ = 4.83, df = 2, *p* = 0.09).

Complete Resection of Index Metastases: Assessing Survival Benefits for Patients with Multiple Metastases

**Figure 2:**
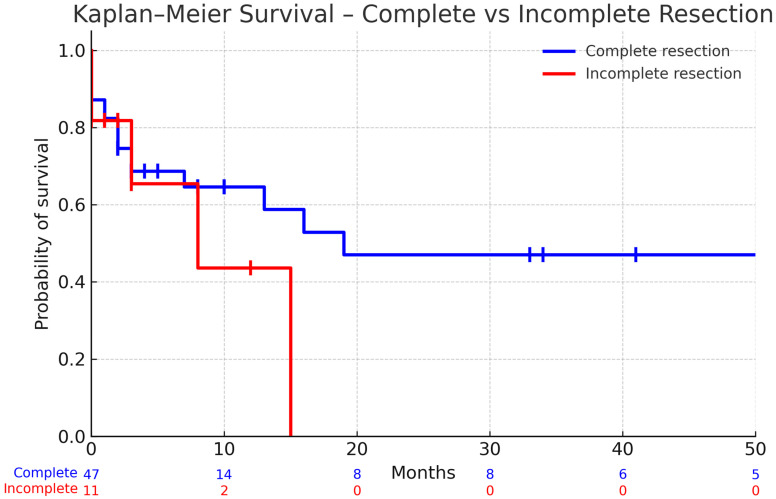
Kaplan–Meier survival according to extent of resection in patients with multiple (>3) metastases.

Figure 2 presents a survival analysis that examines whether achieving complete resection of index metastases improves survival in patients with multiple brain metastases. In patients with multiple metastases, complete resection was associated with longer mean survival (15.74 months, 95% CI 10.22–21.26) compared with incomplete resection (4.5 months, 95% CI 1.29–7.71), though the difference was not statistically significant (*p* = 0.3649). The lack of statistical significance may be attributed to the small sample size, particularly in the incomplete resection group, as suggested by the wide confidence intervals (Figure 2).

Kaplan–Meier survival curves are shown for patients with complete resection (blue) and incomplete resection (red). Numbers at risk are displayed beneath the x-axis at 0, 10, 20, 30, 40, and 50 months. Median survival was not reached in the complete resection group, whereas patients with incomplete resection showed shorter median survival (not beyond 15 months). The log-rank (Mantel–Cox) test did not demonstrate a statistically significant difference between groups (χ^2^ = 0.33, df = 1, *p* = 0.56). The Gehan–Breslow–Wilcoxon test yielded a similar result (χ^2^ = 0.46, df = 1, *p* = 0.50).

### 3.2. Functional Outcome After Surgery

Functional status was assessed using the Karnofsky Performance Status (KPS), which was recorded preoperatively and postoperatively. No additional standardized neurocognitive instruments were applied. The analysis examined changes in KPS before and after surgical resection across three patient groups categorized by the number of brain metastases. In patients with a single metastasis, the median KPS remained stable at 80% (IQR: 70–90%) from pre- to postoperative assessment, with no statistically significant change (*p* = 0.900).

Similarly, patients with oligometastases (2–3 lesions) showed no significant change in KPS, although there was a trend toward a decline from a median of 80% (IQR: 60–90%) preoperatively to 70% (IQR: 50–90%) postoperatively (*p* = 0.123). Patients with multiple metastases (>3) maintained a median KPS of 70% before and after surgery, with interquartile ranges of 60–80% preoperatively and 50–80% postoperatively, approaching but not reaching statistical significance (*p* = 0.070). In addition to mean KPS values, we assessed clinically meaningful changes. A ≥ 10-point decline was observed in 5.2% of patients with a single metastasis (4/77), 8.6% with oligometastatic disease (3/35), and 2.1% with multiple metastases (1/47).

These findings suggest that neurosurgical resection generally preserved functional status across all three groups, without significant improvement or deterioration of KPS scores (Table 2). This stability in performance status is clinically relevant, as maintaining functional independence is a key goal in the management of patients with brain metastases.

### 3.3. Postoperative Complications

The analysis examined whether the number of brain metastases was associated with complications, length of hospitalization, and receipt of adjuvant therapies. Regarding adverse effects, patients with oligometastases showed the highest rate of complications (37.1%) compared with those with multiple metastases (20.8%) and a single metastasis (18.2%). However, this difference did not reach statistical significance (*p* = 0.079).

Length of hospitalization was consistent across all three groups, with a median stay of 10 days for patients with a single metastasis (IQR: 6–17), oligometastases (IQR: 6–15), and multiple metastases (IQR: 6–17). Statistical analysis confirmed no significant difference in hospital stay duration between groups (*p* = 0.943). Postoperative complications were systematically classified according to the Clavien–Dindo system. Within the first 30 days, four patients (2.0%) died, representing the 30-day mortality of the cohort. Clinically relevant adverse events were uncommon. In total, five patients experienced Clavien–Dindo grade IIIb complications, consisting of two postoperative infections requiring revision surgery and three postoperative hemorrhages. Six patients developed grade IV complications; of these, two were postoperative intracranial hemorrhages, whereas the remaining cases were related to non-surgical systemic complications (pulmonary embolism, acute kidney injury, and pulmonary decompensation).

Regarding adjuvant therapies, the rate of postoperative radiation therapy was similar across groups: 63 of 77 patients (81.8%) with a single metastasis, 25 of 35 patients (71.4%) with oligometastases, and 38 of 48 patients (79.2%) with multiple metastases (*p* = 0.459). For postoperative systemic therapy, patients with a single metastasis had the highest rate, with 59 of 77 patients (76.6%), compared with 20 of 35 patients (57.1%) in the oligometastatic group and 29 of 48 patients (60.4%) in the multiple metastases group. This difference approached but did not reach statistical significance (*p* = 0.057).

These findings suggest that the number of brain metastases does not significantly impact the occurrence of surgical complications, length of hospitalization, or receipt of adjuvant therapies. However, the near-significant trends observed for adverse effects and postoperative systemic therapy warrant further investigation with larger sample sizes.

The analysis examined potential associations between various clinical factors and the occurrence of postoperative epileptic seizures in patients who underwent surgical resection for brain metastases. All patients in the cohort experienced seizures classified as “early”, occurring within 30 days post-surgery. Among the demographic and clinical characteristics, sex showed no significant association with postoperative seizures (*p* = 0.231), though females had a slightly higher representation in the seizure group (53.7%) compared with the non-seizure group (42.9%). Moreover, seizure patients were slightly older (median 70 years, IQR 55–77) than non-seizure patients (median 66 years, IQR 56–74). Patients with multiple metastases experienced significantly more postoperative seizures compared with single lesions (OR = 0.21, 95% CI 0.09–0.49, *p* = 0.0003) and oligometastatic disease (OR = 0.28, 95% CI 0.10–0.78, *p* = 0.019). No difference was observed between single and oligometastatic disease (OR = 0.96, 95% CI 0.35–2.61, *p* = 1.0).

**Figure 3:**
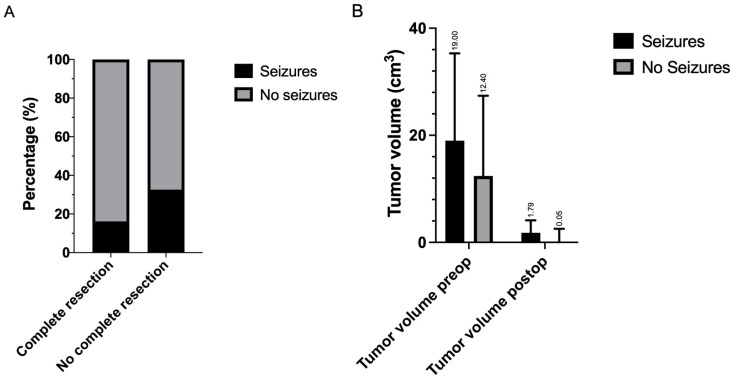
(**A**) Association of postoperative seizures with complete cytoreduction. (**B**) Association of preoperative and postoperative seizures with tumor volume.

Patients who did not achieve complete resection had a substantially higher risk of seizures (32.6%) compared with those with complete resection (16.2%). This corresponded to an odds ratio of 2.53 (95% CI 1.14–5.64, *p* = 0.019, Figure 3A). Neither postoperative radiation (*p* = 0.311) nor postoperative systemic therapy (*p* = 0.301) showed significant associations with seizure occurrence.

Patients with seizures had larger postoperative residual tumor volumes (median 1.79 vs. 0.05 cm^3^, *p* = 0.0065; Cliff’s δ = 0.28, 95% CI 0.08–0.46). Preoperative volumes were also higher (median 19.0 vs. 12.3 cm^3^, *p* = 0.024; Cliff’s δ = 0.24, 95% CI 0.02–0.44), although this narrowly missed Bonferroni-adjusted significance (Figure 3B).

These findings suggest that incomplete resection and higher residual tumor burden are significant risk factors for postoperative seizures.

## 4. Discussion

Despite advancements in neuro-oncological treatments, the optimal management of patients with multiple brain metastases remains a subject of intense investigation and debate. While surgical intervention, radiosurgery, and systemic therapies have all contributed to improved outcomes, determining the best approach for patients harboring numerous lesions continues to challenge clinicians. The study of surgical resection still evokes questions regarding the balance between potential survival benefits and the risks of postoperative complications. Consequently, this necessitates careful consideration of individual patient factors, such as tumor burden, systemic disease status, and neurological functionality, to tailor the most effective treatment strategy [23,24,25].

Resection of brain metastases followed by radiotherapy can significantly improve patient prognosis, enhancing both local control and overall survival [26]. The data from our study suggest that when complete resection of the index metastasis is achieved, the addition of radiotherapy to manage the remaining metastatic lesions may not significantly alter overall survival outcomes. While traditional approaches have emphasized the need for extensive surgical intervention to maximize survival, our findings invite a re-evaluation of treatment paradigms, suggesting that a strategic combination of surgery and targeted radiotherapy might suffice in optimizing patient outcomes.

In light of these findings, alternative treatment strategies such as radiosurgery offer less invasive options that have shown promise in managing both solitary and multiple lesions. SRS, which delivers targeted radiation to specific lesions, may provide benefits comparable to surgical approaches, particularly for patients who cannot undergo surgery or those with fewer, smaller metastases. Comparative studies suggest that SRS alone or in conjunction with surgery may offer similar survival benefits with a potentially lower risk of complications [27,28,29,30]. However, patient selection remains critical to maximizing the efficacy of SRS, necessitating a tailored approach based on comprehensive evaluations.

Recent advances in systemic therapies have significantly altered the management landscape for patients with brain metastases, impacting survival outcomes. The introduction of third-generation tyrosine kinase inhibitors has demonstrated intracranial response rates exceeding 80%, offering a powerful systemic treatment option for patients with metastatic cancer involving the brain [31,32]. These inhibitors, known for their ability to penetrate the central nervous system, have broadened the treatment repertoire, complementing traditional radiotherapy and surgical approaches.

Recent clinical and surgical series provide increasing support for the role of aggressive local therapy in brain metastases. A large institutional series published in 2024 reported that gross total resection was achieved in 87.2% of lesions; 42.6% of patients improved in KPS postoperatively, 46.8% remained unchanged, and 10.6% worsened, with a 30-day mortality of 2.1% [33]. These results echo our observation that functional status may be preserved in the majority of treated patients. Similarly, a recent systematic review confirmed that more complete surgical removal is associated with improved prognosis in many cohorts [34]. Our study further supports the broader literature, which advocates thorough tumor resection, aligning with evidence that aggressive cytoreduction prolongs survival [35,36]. Although statistical significance was not reached, likely due to sample size limitations, the trend supports the practice of striving for maximal cytoreduction to optimize outcomes [17]. While our data suggest a trend toward longer survival following complete resection in patients with multiple metastases, this finding did not reach statistical significance. It should therefore be interpreted cautiously and viewed as hypothesis-generating rather than confirmatory.

The stability of functional outcomes post-surgery, as measured by KPS, attests to the surgical preservation of patient quality of life [37]. Our findings suggest that neurosurgical resection generally preserved functional status without significant improvement or deterioration of KPS scores. While the overall analysis showed stability in functional outcomes post-surgery across the different metastasis groups, the nearly significant *p*-values for changes in KPS in patients with oligometastatic and multiple metastases suggest a trend toward functional decline. This indicates that although surgical intervention typically preserves functional independence, patients in these categories may experience subtle declines in functionality that are worth monitoring. This potential decline underlines the necessity for individualized postoperative management plans and highlights the importance of further research to confirm these trends and develop strategies to mitigate functional deterioration in more complex cases of brain metastases.

Our study found that patients with brain metastases experience postoperative complications, particularly when complete resection is not achieved, aligning with findings that the presence of multiple intracranial lesions increases the likelihood of adverse events following surgical intervention [38]. Furthermore, increased tumor burden correlates with higher postoperative risk, underscoring the importance of maximizing tumor resection during surgery. Proper preoperative management is crucial for ensuring safe resection of brain metastases, as it helps to mitigate complications and optimize surgical outcomes [39]. The ability to achieve complete resection was associated with fewer postoperative seizures, suggesting an advantage in focusing on maximal reduction in tumor burden [11].

With respect to seizure outcomes, a retrospective analysis demonstrated that larger tumor volume, greater necrosis-to-tumor ratio, and midline shift were significant predictors of persistent postoperative seizures in patients with brain metastases [40]. Together, these studies corroborate key aspects of our results—particularly the importance of resection extent and tumor burden—while our work adds further nuance by focusing on the subset of patients with multiple metastases treated via index lesion resection plus adjuvant radiotherapy. Postoperative seizure risk correlates with residual tumor burden, consistent with previous studies highlighting the need for comprehensive resections to minimize neurological complications [41]. The resultant data reinforce the strategy of achieving total cytoreduction to prevent seizures and improve overall neurologic health post-surgery [42].

While our study provides valuable insights into the impact of surgical resection on patients with multiple brain metastases, several limitations should be acknowledged. First, the sample size, although comprehensive for a single-center study, limits the ability to generalize findings broadly and may reduce the statistical power of subgroup analyses, particularly when assessing the survival benefits associated with complete resection. Additionally, as a retrospective study, there is inherent selection bias regarding which patients received surgical intervention versus other treatments. Specifically, patients chosen for surgery may have had more favorable clinical characteristics, potentially skewing results. Finally, the absence of a control group of non-surgical patients restricts our ability to definitively attribute the observed outcomes solely to surgical intervention. Future studies with larger, multicenter cohorts and robust control groups will be invaluable in validating these findings and refining treatment strategies for effectively managing brain metastases.

In summary, our study supports the integrated approach of aggressive surgical management complemented by adjuvant therapies, proving crucial for favorable outcomes in patients with multiple brain metastases. This study is limited by its retrospective design, which introduces potential selection bias in determining surgical candidacy. Furthermore, although adjuvant radiotherapy was administered in the majority of cases, detailed treatment parameters were not available for all patients, particularly those treated externally. This limits the generalizability of our findings and highlights the need for prospective studies with standardized documentation of adjuvant therapies. Although effect sizes and confidence intervals were reported wherever feasible, in some analyses (particularly those with very small subgroups) these could not be reliably calculated and were therefore not provided. We acknowledge this as a limitation of our study design and sample size. Given the statistical limitations, including the sample size and lack of a control group, we advise caution in declaring a paradigm shift. The findings suggest potential advantages of aggressive surgical management combined with adjuvant therapies, but these results should primarily serve as a basis for generating hypotheses in future studies. Further research, ideally with larger sample sizes and diverse cohorts, is necessary to validate these initial observations and refine treatment strategies. Future studies should systematically analyze combinations of adjuvant therapies with surgery. This would enable a clearer understanding of how different treatment regimens influence outcomes. Including non-surgical control groups could offer direct comparisons, helping to isolate the effects of surgical resection from other treatment modalities. Integrating molecular profiling and biomarker analyses could identify patients most likely to benefit from specific interventions, facilitating more personalized treatment approaches. By prioritizing comprehensive investigations, we can better understand the nuanced impacts of treatment combinations and their roles in improving patient outcomes in neuro-oncology.

## 5. Conclusions

Aggressive surgery combined with adjuvant therapy improved survival and functional outcomes in selected patients with multiple brain metastases. Notably, the number of brain metastases did not significantly impact survival outcomes when complete resection of index metastasis and adjuvant radiation were performed, challenging traditional expectations of poorer prognosis with multiple lesions.

Functional status as measured by KPS remained generally stable across all metastasis groups following surgery, indicating that neurosurgical intervention successfully preserved neurological function. Regarding complications, the number of brain metastases was not significantly associated with adverse effects, length of hospitalization, or receipt of adjuvant therapies. Risk factors for postoperative seizures included incomplete resection of all brain metastases and a higher residual tumor volume, highlighting the importance of maximal safe resection in seizure prevention.

These findings suggest that aggressive surgical management of index metastases, when combined with appropriate adjuvant therapy, can yield favorable outcomes regardless of the total number of brain metastases.

## Figures and Tables

**Table 1:** Demographic and clinical characteristics of patients undergoing surgical resection for brain metastases.

Variable	Category	N (%)
Sex	Male	87 (54.4)
	Female	73 (45.6)
Age, years	Median (IQR, range)	67 (56–74, 20–90)
History of epileptic seizures before surgery	Yes	27 (16.9)
	No	133 (83.1)
History of epileptic seizures after surgery	Yes	41 (25.6)
	No	119 (74.4)
Extracranial disease	Metachronous	64 (40.0)
	Synchronous	96 (60.0)
Primary tumor histology	Lung	53 (33.1)
	Breast	14 (8.8)
	Melanoma	24 (15.0)
	CUP	5 (3.1)
	Gastrointestinal (GI)	15 (9.4)
	Kidney	12 (7.5)
	Urothelial	5 (3.1)
	Other	32 (20.0)
No. of brain metastases	Single metastasis	77 (48.1)
	Oligometastases (2–3)	35 (21.9)
	Multiple (>3)	48 (30.0)
Complete resection of index metastasis	Yes	134 (83.8)
	No	26 (16.3)
Total number of metastases	Median (IQR, range)	2 (1–4, 1–16)
Total tumor volume (cm^3^)	Median (IQR)	14 (7–31)
Volume of resected lesion (cm^3^)	Median (IQR)	12 (6–26)
Preoperative KPS	Median (IQR)	80 (60–90)
Postoperative KPS	Median (IQR)	80 (60–90)
Hospital stay, days	Median (IQR, range)	10 (6–17, 3–74)
Adverse effects (Clavien–Dindo grade)	Any adverse effect	37 (23.1)
	Grade I	13 (35.1)
	Grade II	2 (5.4)
	Grade IIIa	1 (2.7)
	Grade IIIb	5 (13.5)
	Grade IVb	7 (18.9)
	Grade V	9 (24.3)
Radiation therapy (post- or intraoperatively)	Yes	135 (84.4)
	No	25 (15.6)
Systemic therapy postoperatively	Yes	108 (67.5)
	No	52 (32.5)

**Table 2:** Karnofsky Performance Status (%) before and after surgery by metastasis group.

Patient Group	Preoperative KPS Median (IQR), Mean Rank	Postoperative KPS Median (IQR), Mean Rank	*p*-Value
Single metastasis	80 (70–90), 15.91	80 (70–90), 17.17	0.900
Oligometastases	80 (60–90), 14.50	70 (50–90), 7.15	0.123
Multiple metastases	70 (60–80), 12.93	70 (50–80), 9.00	0.070

KPS—Karnofsky Performance Status. Mean ranks based on Wilcoxon signed-rank test comparisons (postoperative vs. preoperative).

## Data Availability

All relevant data are included within the manuscript. Any additional data that support the findings of this study are available upon reasonable request from the corresponding author.
